# Multidirectional therapeutic effects of synthesized HMGB1 peptide on liver cirrhosis in mice

**DOI:** 10.1016/j.bbrep.2025.102061

**Published:** 2025-05-25

**Authors:** Masaki Mito, Atsunori Tsuchiya, Soichi Ishii, Takafumi Tonouchi, Kaito Furuyama, Ryo Jinbo, Nobutaka Takeda, Hiroyuki Abe, Katsuto Tamai, Shuji Terai

**Affiliations:** a Division of Gastroenterology and Hepatology, Graduate School of Medical and Dental Sciences, Niigata University, 1-757, Asahimachi-dori, Chuo-ku, Niigata, 951-8510, Japan; b Department of Stem Cell Therapy Science, Graduate School of Medicine, Osaka University, 2-2, Yamadaoka, Suita, Osaka, 565-0871, Japan; c StemRIM Institute of Regeneration-Inducing Medicine, Osaka University, 2-8, Yamadaoka, Suita, Osaka, 565-0871, Japan

**Keywords:** HMGB1, Peptide, Liver cirrhosis, Spatial transcriptomics analysis, Fibrosis

## Abstract

**Aim:**

Liver cirrhosis is a serious disease characterized by liver dysfunction and severe fibrosis; however, no breakthrough drugs have effectively improved fibrosis, making it an unmet medical need. We have previously reported that the HMGB1 peptide, synthesized from box A of the HMGB1 protein, ameliorates liver fibrosis and is a promising candidate for fibrosis-improving drugs against liver cirrhosis. In this study, we used spatial analysis to observe treatment-induced changes over time.

**Methods:**

Liver cirrhosis was induced in C57BL/6J mice using carbon tetrachloride (CCl4) injections, followed by HMGB1 peptide treatment. To assess the temporal effects of HMGB1 on the liver in a CCl4-induced cirrhosis mouse model, we used GeoMx spatial analysis. We focused on αSMA-positive active hepatic stellate cells (HSCs), F4/80-positive macrophages, and CK8/18-positive hepatocytes to determine how each cell type was affected over time. Statistical analyses were conducted using GraphPad Prism9, with significance set at p < 0.05.

**Results:**

In cirrhotic mice, we first observed a decrease in the number of activated HSCs over time, two weeks after treatment initiation. Macrophage-associated genes ceased to induce fibrosis-related pathways early in the treatment. This suggests that the effect of macrophages on fibrosis was weakened by the treatment. We also confirmed that lipid metabolism of hepatocytes may be improved during treatment. Furthermore, *Cxcl12* and *Ccl25* expression were induced in the peptide-treated group, indicating possible cell migration to the liver.

**Conclusion:**

Over time, macrophages followed by HSCs, showed the most notable changes with treatment, resulting in improved fibrosis. The HMGB1 peptide drug also affected lipid metabolism in hepatocytes, suggesting a positive therapeutic effect on steatohepatitis. Elevated factors that promote cell migration may have also enhanced the healing effect.

## Highlights

• HMGB1 peptide treatment reduces αSMA-positive cells from week two in cirrhosis model.
• Control group shows collagen pathway activity; not seen in treated group.
• Treatment enhances lipid metabolism in hepatocytes.
• GPCR ligand binding detected in both macrophages and hepatocytes.
• Treatment may reduce liver injury and fibrosis via multiple pathways.

## 1 Introduction

Cirrhosis is formed by fibrosis following chronic liver damage caused by viruses or steatohepatitis due to alcohol or dietary factors. The degree of fibrosis in liver disease is directly related to prognosis, with advanced cirrhosis causing various effects, such as jaundice, ascites, hepatic encephalopathy, and ruptured varices due to decreased liver function and portal hypertension [1,2]. Liver transplantation is the only life-saving option in such cases. Therefore, fibrosis-improving drugs are an unmet medical need in cirrhosis treatment, representing a promising area of development [3].

The HMGB1 peptide synthesized from Box A of HMGB1 was used in this experiment. It is a candidate substance that has been reported to improve liver fibrosis in basic research [4,5]. HMGB1 has two regions, Box A and Box B, and plays two opposing roles. Box B is known to induce tissue remodeling and activate inflammatory responses by binding to Toll-like receptors (TLRs) and/or advanced glycation end-product receptors on the surface of target cells. On the other hand, Box A of HMGB1 is known to prevent inflammation from spreading to surrounding tissues [4]. This Box A-derived HMGB1 peptide was developed by Tamai et al. for epidermolysis bullosa. They reported that the drug exerts its effects through the involvement of mesenchymal stem cells (MSCs) in the body [6,7], and further research is currently underway into the mechanism [8,9].

We have previously shown that HMGB1 peptide reduces liver injury and improves fibrosis in a cirrhosis model induced by carbon tetrachloride (CCl4) [4]. We also demonstrated short-term improvement in liver injury, fat metabolism, and fibrosis as well as long-term improvement in fibrosis and reduction in tumorigenesis in melanocortin-4 receptor-deficient (Mc4r-KO) mice, a model of metabolic dysfunction-associated steatohepatitis (MASH) [5]. Investigator-initiated clinical trials using this peptide are currently underway for patients with chronic liver disease.

In this study, we utilized GeoMx spatial analysis to analyze the effects of HMGB1 peptide on the liver, focusing on changes in three cell types: α smooth muscle actin (SMA) positive activated hepatic stellate cells (HSCs), F4/80-positive macrophages, and CK8/18-positive hepatocytes. We aimed to follow the temporal changes in these cells post-treatment, analyzing modifications in the liver induced by administration of the peptide.

## 2 Methods

### 2.1 Mice

C57BL/6J male mice purchased from Charles River (Yokohama, Japan) were used. All of the animal experiments were approved by the Niigata University Review Board for Animal Care (approved number; SA01239). This study was reported in accordance with the ARRIVE 2.0 Essential 10 guidelines (https://arriveguidelines.org). All animals were housed in a specific pathogen-free environment and maintained under standard conditions with a 12-h day-night cycle and *ad libitum* access to food and water.

### 2.2 HMGB1 peptide

HMGB1 peptide (S-005151) was provided by Shionogi & Co., Ltd. (Osaka, Japan). This peptide consists of amino acids 1 to 44 of the HMGB1 protein and contains the KOI2 domain, also known as the MSC induction domain, present at the N-terminus of Box A of HMGB1. Thus, this peptide does not include Box B, which has pro-inflammatory effects. The HMGB1 peptide was dissolved in normal saline (NS, 1 mg/mL; Otsuka Pharmaceutical Co., Ltd., Tokyo, Japan) before injection [4,5].

### 2.3 Liver cirrhosis model preparation and treatment

**Fig. 1:**
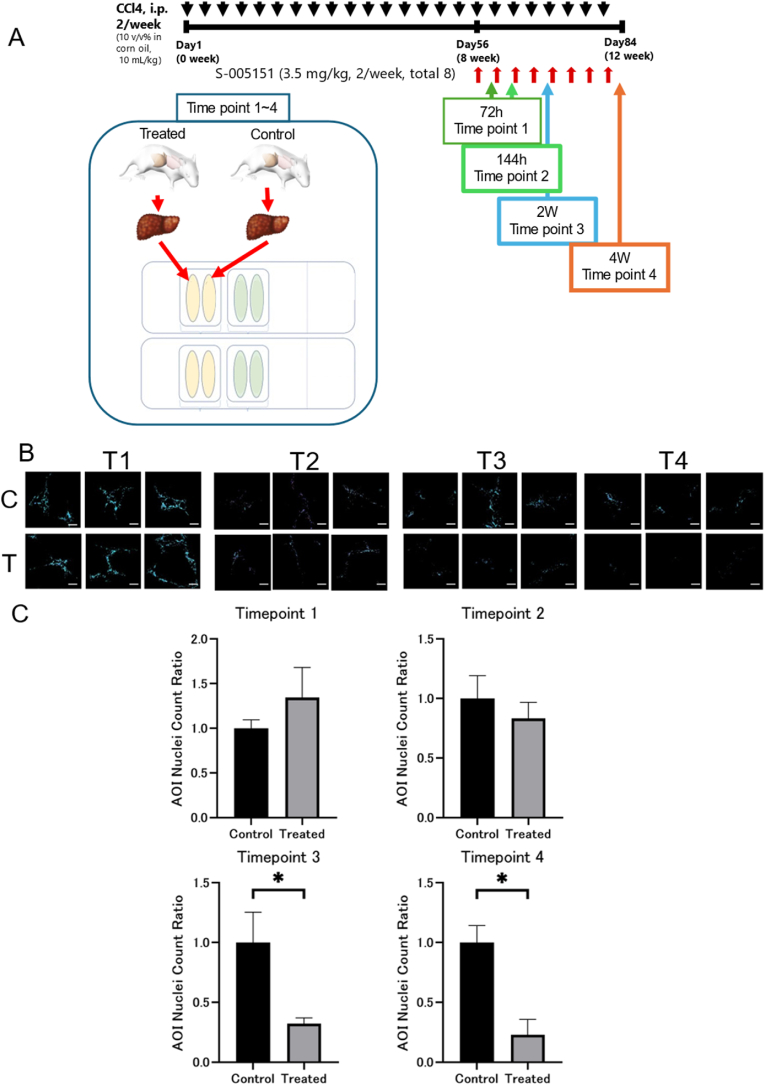
**Time schedule of the complete animal experiment and temporal changes in αSMA following treatment.** (A) Induction of liver cirrhosis, drug administration intervals, and analysis time points. (B) Temporal changes in αSMA immunostaining. (C) Differences in the number of αSMA-positive nuclei between treated and non-treated (control) groups over time. c; control, T; treated (n = 3 in each group). The data is shown as means ± SEMs. ∗*p <* 0.05. Scale bars = 100 μm.

Eight-weeks-old male mice were intraperitoneally injected twice a week with 1.0 mL/kg CCl4 (FUJIFILM Wako Pure Chemical Corporation, Osaka, Japan) dissolved in corn oil (1:10 v/v; FUJIFILM Wako Pure Chemical Corporation) for 12 weeks to induce cirrhosis [10]. Eight weeks after CCl4 injection, HMGB1 peptide (3.5 mg/kg) or NS (control) was also administered via the tail vein twice a week for 4 weeks. Mice were sacrificed 72 h (time point 1), 144 h (time point 2), 2 weeks (time point 3), and 4 weeks (time point 4) after initiating HMGB1 peptide administration. At each time point, three mice were created for each control and treatment group, and one mouse from each time point was selected for analysis. Their livers were removed and used to prepare tissue samples for spatial analysis (Fig. 1A).

### 2.4 Spatial transcriptomics analysis by GeoMx

**Fig. 2:**
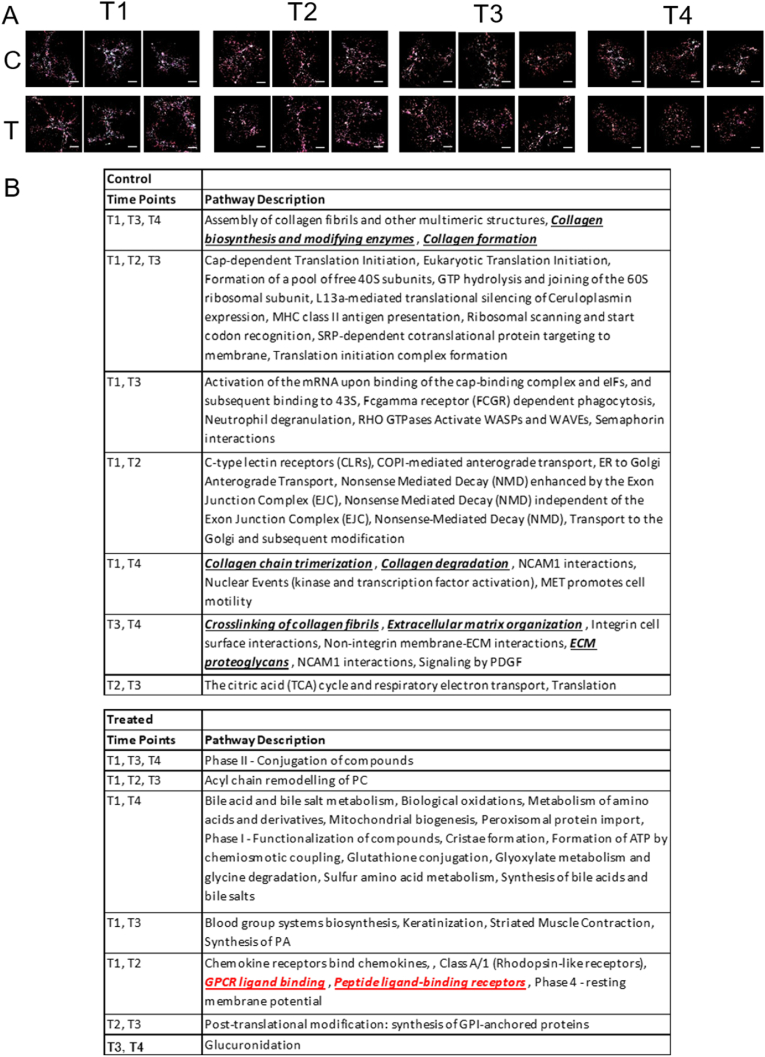
**Temporal changes in macrophages following treatment.** (A) Macrophage immunostaining results. (B) Items detected across >3 or 2 points per time point in a pathway analysis of control and treated mice. Scale bars = 100 μm.

**Fig. 3:**
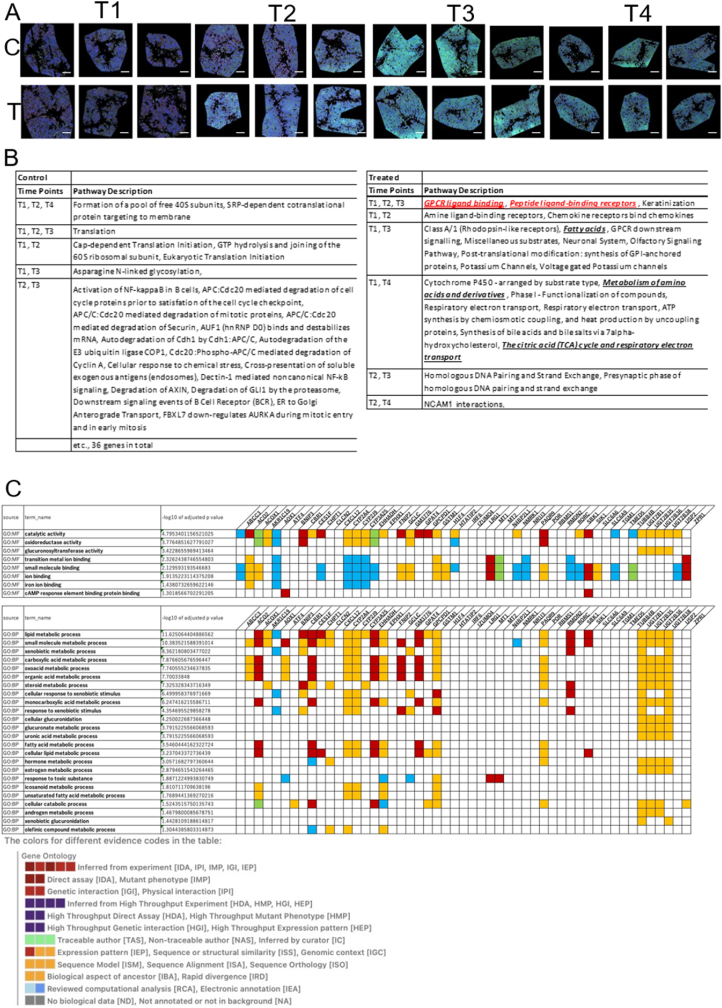
**Temporal changes in hepatocytes following treatment.** (A) Hepatocyte immunostaining results. (B) Items detected across >3 or 2 points per time point in a pathway analysis of control and treated mice. (C) Pathway analysis only for mRNA with high expression or significant difference (X-axis Log2 > 1 or Y-axis -log10 p-value >2) in the volcano plot. Scale bars = 100 μm.

The spatial analysis followed the NanoString (Seattle, WA, USA) protocol [11,12]. Briefly, samples of liver tissue were prepared and compared between the group treated with HMGB1 peptide and the control group (eight tissue samples in total) at four time points. The tissues were arranged on two slides: the first slide displayed the treated and control tissues at time points 1 and 2, and the second slide displayed those at time points 3 and 4. In the GeoMx analysis, three types of immunostaining were used to collect mRNA from the target cells for analysis. These target cells were α-smooth muscle (αSMA)-positive activated HSCs (myofibroblast), F4/80-positive macrophages, and CK8/18-positive hepatocytes (Fig. 1, Fig. 2, Fig. 3A). The effects of each cell were analyzed at the four time points in both the treated and control groups. For slide preparation, a high-plex mixture of a photocleavable oligo-binding probe (showing RNA) and a morphology reagent were applied to the tissue sections. The slides were loaded into the GeoMx instrument, followed by a series of automated steps. From the immunostained slides, the region of interest (ROI) to be analyzed was selected. Since fibrosis was the main target of this study, ROIs were selected such that they were centered on the area of fibrosis and had approximately the same area. Three ROIs were created for each of the eight liver tissues. The GeoMx device was then used to irradiate each area of interest with UV light. The released oligos were collected and precipitated on a microtiter plate. Thus, photocleaved oligos of the three target cell types were obtained from each ROI. Finally, next-generation sequencing was used to obtain and analyze RNA information for each gene. Pathway analysis and volcano plots were created using DSP GeoMx software (NanoString, Seattle, WA, USA) [[13], [14], [15]]. For further gene analysis, g-profiler (Institute of Computer Science University of Tartu, Tartu, Estonia) was also used.

### 2.5 Statistical analyses

Statistical analyses were performed using DSP GeoMx software and GraphPad Prism9 software (GraphPad Software Inc., La Jolla, CA, USA). Data (the number of nuclei in the ROIs of αSMA-positive cells) are presented as means ± SEMs. The results were statistically analyzed using the Mann–Whitney *U* test. Differences were considered significant at *p <* 0.05.

## 3 Results

### 3.1 Number of activated hepatic stellate cells decreases over time in cirrhotic mice

Immunostaining showed a decrease in the stained area of activated HSCs (αSMA-positive cells) in the peptide-treated group starting 2 weeks after treatment initiation (Fig. 1B). Consistent with these results, the number of nuclei in the ROIs was also significantly reduced (Fig. 1C). These results indicate that the peptide decreases the number of activated HSCs over time in cirrhotic mice.

### 3.2 Fibrosis-related pathways are inhibited in macrophages from early post-treatment

In a pathway analysis of F4/80-positive macrophages over time, the common pathways were identified and summarized. Collagen biosynthesis and modifying enzymes (time points 1, 3, and 4) collagen formation were detected thrice in the control group alone. Meanwhile, collagen chain trimerization (time points 1 and 4), collagen degradation (time points 1 and 4), crosslinking of collagen fibrils (time points 3 and 4), extracellular matrix (ECM) proteoglycans (time points 3 and 4), and extracellular matrix organization (time points 3 and 4) were detected twice (Fig. 2B). None of these were detected more than once in the treatment groups (Fig. 2C), suggesting that macrophages after treatment do not promote fibrosis.

### 3.3 Hepatocytes may be altered in lipid metabolism

To determine the effect of the peptide on CK8/18-positive hepatocytes, a temporal pathway analysis was performed, as with macrophages, to extract and summarize the common pathways. In the treatment group, items related to fatty acid metabolism (time points 1 and 3), metabolism of amino acids and derivatives (time points 1 and 4), and the citric acid cycle with respiratory electron transport (time points 1 and 4) were extracted (Fig. 3B and C). For both the control and treatment groups, genes that appeared more than twice at each time point in the volcano plot and met the criteria (Log2 ≥ 1 on the X-axis or a -log10 p-value ≥2 on the Y-axis) were extracted. The extracted genes were placed in a g: Profiler and subjected to Gene Ontology (GO) analysis. Catalytic activity was the most significant molecular function, while lipid metabolism was the most significant biological process. Genes such as *Acox1*, *Cbr1*, *Ces1f*, *Chpt1*, *Ehhadh*, *Gpat4*, *Rorc*, and *Sik1* were involved in lipid metabolism (Fig. 3C). These results suggest that the peptide treatment induces changes in lipid metabolism and other metabolic systems in hepatocytes.

### 3.4 *Prlh, Cxcl12* and *Ccl25* are induced in the livers of the peptide-treated group

**Fig. 4:**
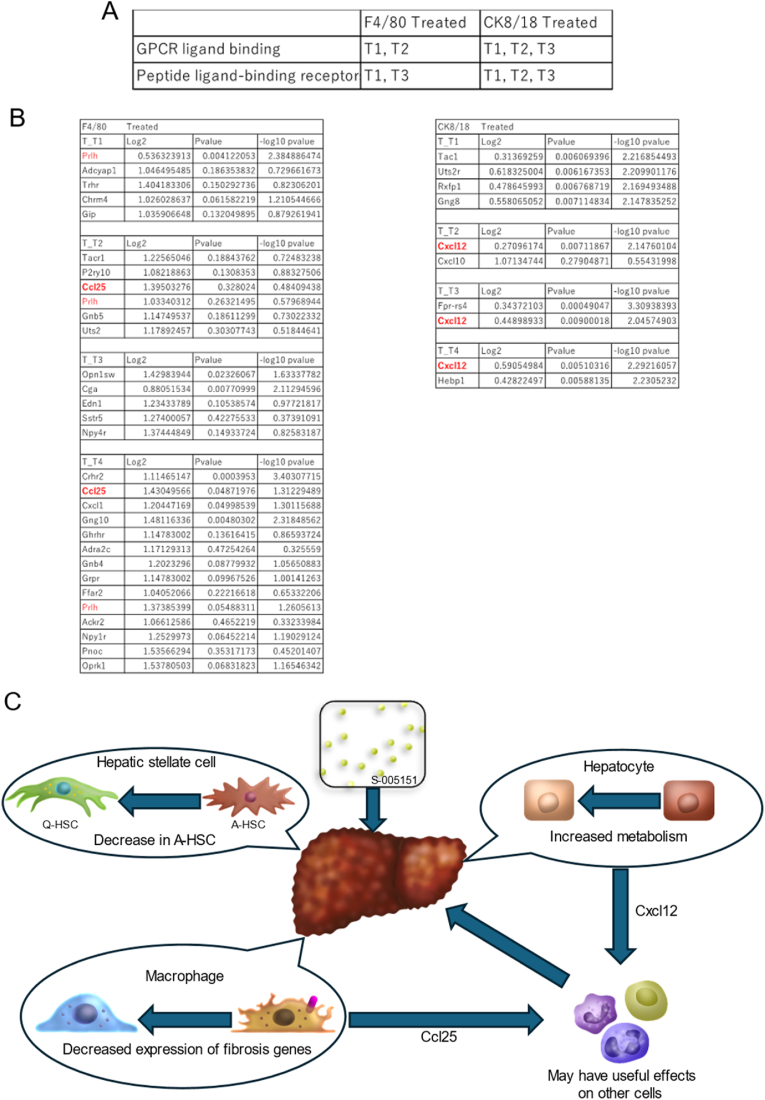
**Commonly detected pathways between cells and summary of results.** (A) Common pathway items identified more than once across different cell types. (B) Genes related to commonly recognized pathways that are highly expressed or have significant differential expression (Log2 on the X-axis >1 or -log10 p-value on the Y-axis >2) in the volcano plot. (C) Summary of results.

Next, we identified the common treatment effects across cell types: G-Protein Coupled Receptors (GPCR) and peptide ligand-binding receptors were observed at two time points for macrophages and at three time points for hepatocytes in the treatment group (Fig. 4A). These changes were thought to be treatment-induced, revealing common pathway genes among those with X-axis Log2 values > 1 or Y-axis -log10 p-values >2. *Prlh* (time points 1, 2, and 4) and *Ccl25* (time points 2 and 4) were extracted from the macrophages. In hepatocytes, *Cxcl12* (timepoint 2, 3, 4) was extracted (Fig. 4B).

## 4 Discussion

In this study, we compared changes in αSMA-positive (activated HSCs), F4/80-positive (Macrophages), and CK8/18-positive (hepatocytes) cells in the control and treatment groups over time. We used GeoMx spatial analysis on a cirrhosis mouse model created using CCl4 and treated with HMGB1 peptide synthesized from Box A of HMGB1. The number of αSMA-positive cells was significantly reduced in the treated group following the second week after treatment. In F4/80-positive macrophages, multiple pathways related to collagen-containing extracellular matrix and its cross-linking were detected in the control group, but not in the treatment group. In the hepatocytes, changes in lipid metabolism were observed in the treatment group. In addition, pathways such as GPCR ligand-binding were commonly detected in macrophages and hepatocytes. A more detailed analysis of the genes found in these pathways revealed factors such as CCl25 and CXCL12, which are involved in cell migration. Overall, we found that the treatment had a positive effect on the cells in multiple ways, including improvement of fibrosis and lipid metabolism, as well as reduction of inflammation (Fig. 4C). These results support our previously published findings of reduced liver injury in a cirrhosis mouse model, as well as improved lipidosis, reduced liver injury, and reduced fibrosis in a MASH mouse model.

In a previous single-cell RNA analysis of the hematopoietic system of the liver tissue, 4 weeks after the administration of HMGB1 peptide, macrophages were the most altered cells [4]. This is consistent with our observations in the CCl4 mouse model. Additionally, AST and ALT levels decreased in the first week of administration, followed by an improvement in fibrosis two weeks later. Again, the changes in macrophages were seen before the changes in the number of αSMA-positive cells, which potentially indicates a timeline in which fibrosis improves after liver injury or inflammation is reduced. Later the timepoint, more frequent the detection of pathways and components involved in fibrosis enhancement, including crosslinking of collagen fibrils (time points 3 and 4), ECM proteoglycans (time points 3 and 4), and extracellular matrix organization (time points 3 and 4). This suggests that differences in fibrosis firmness become more apparent over time. Considering that the direct effects of this peptide on macrophages and hepatic stellate cells were minor in our previous study [4], they may be attributed to in vivo induction of cells or substances in the liver post administration, which subsequently affected the HSCs. It will be interesting to see how this drug affects macrophages in vivo in the future.

In our previous study on a MASH mouse model, we demonstrated short-term improvements in liver injury, fat metabolism, and fibrosis, as well as long-term improvements in fibrosis and reduced tumorigenesis. Lipidomics revealed lipolysis promotion owing to activation of fatty acid β-oxidation and improvement in insulin resistance [5]. The mouse model used in the present study was a CCl4 model, and the lipid metabolic process was the first to be altered in hepatocytes. It is not clear whether this is due to the normalization of hepatocytes as a result of improved liver damage or an effect unrelated to liver damage; however, the results indicate a particularly strong effect of this drug on lipid metabolism. The genes of interest included *Acox1*, *Cbr1*, *Ces1f*, *Chpt1*, *Ehhadh*, *Gpat4*, *Rorc*, and *Sik1*. ACOX1 is the first enzyme in the fatty acid β-oxidation pathway to catalyze the unsaturation of acyl-CoA to 2-trans-enoyl-CoA, which is highly relevant to our previous conclusion that it promotes fatty acid β-oxidation [16]. CBR1 has a key role in the DNA damage response through the regulation of IR-mediated ROS generation, and gene expression may be involved in ROS mitigation [17]. In addition, studies using *Ces1*−/− background (TgCES1) have reported that TgCES1 mice display increased triglyceride secretion from the liver to the plasma, together with higher triglyceride levels in the male liver [18]. These results indicate that the carboxylesterase 1 family plays essential roles in drug and lipid metabolism and detoxification.

Thus, these genes are involved in lipid metabolic processes and ROS reduction, again suggesting that they may affect MASH. It will be of considerable interest to determine whether these genetic changes are a direct effect of the drugs, or simply improve function as liver damage is reduced.

Notably, both GPCR and peptide ligand-binding receptors were detected in macrophages and hepatocytes through pathway analysis. Further analysis revealed that *Prlh* and *Ccl25* in macrophages are commonly identified as genes with elevated *Cxcl12* expression in hepatocytes.

The present study did not find much significance for *Prlh*, whereas both *Ccl25* and *Cxcl12* were involved in cell migration. CCL25 is important for MSC accumulation and knee osteoarthritis. In this study, the authors discovered that the chemokine CCL25 is a chemoattractant for human MSC, that is, CCL25-guided in vitro MSC mobilization is increased more than tenfold compared to that with CXCL12 [19,20].

CXCL12, also known as SDF-1, has been the subject of numerous studies because of its involvement in MSC migration [21]. It is strongly expressed in cholangiocytes in the liver [22], and its continuity with hepatocytes may have confirmed this in the analysis. This type of cell migration could have possibly affected MSCs and other immune cells in the liver, thereby promoting healing.

One limitation of this analysis is that it is not clear which cells were directly and indirectly affected by the peptide. However, we believe that we have identified a mechanism by which the HMGB1 peptide can reduce liver injury and ameliorate fibrosis in both cirrhosis and MASH liver models through multiple effects. Fibrosis-improving drugs are an unmet medical need in the field of cirrhosis treatment and we believe that this peptide is a promising agent.

## CRediT authorship contribution statement

**Masaki Mito:** Writing – review & editing, Writing – original draft, Investigation, Formal analysis, Data curation. **Atsunori Tsuchiya:** Writing – review & editing, Writing – original draft, Investigation, Funding acquisition, Formal analysis, Data curation, Conceptualization. **Soichi Ishii:** Data curation. **Takafumi Tonouchi:** Data curation. **Kaito Furuyama:** Data curation. **Ryo Jinbo:** Data curation. **Nobutaka Takeda:** Data curation. **Hiroyuki Abe:** Data curation. **Katsuto Tamai:** Supervision. **Shuji Terai:** Supervision.

## Ethical approval and consent to participate

All animal experiments were conducted in compliance with institutional regulations, and the study protocols were approved by the Institutional Animal Care and Committee of Niigata University.

## Consent for publication

N/A.

## Availability of supporting data

Additional data related to this paper may be requested from the authors.

## Funding

This research was supported by Shionogi & Co., Ltd and StemRIM.

## Declaration of competing interest

The authors declare the following financial interests/personal relationships which may be considered as potential competing interests:Katsuto Tamai reports financial support was provided by StemRIM. Shuji Terai reports a relationship with Shionogi and Co Ltd that includes: funding grants. Atsunori Tsuchiya reports a relationship with Shionogi and Co Ltd that includes: funding grants. Shuji Terai reports a relationship with StemRIM that includes: funding grants. If there are other authors, they declare that they have no known competing financial interests or personal relationships that could have appeared to influence the work reported in this paper.

## Data Availability

The authors do not have permission to share data.

## Abbreviations:

MSC: mesenchymal stem cell
HMGB1: high mobility group box 1
CCl4: carbon tetrachloride
HSC: hepatic stellate cells
SMA: smooth muscle actin
TLR: Toll-like receptor
MASH: metabolic dysfunction-associated steatohepatitis
ROI: region of interest
ECM: extracellular matrix
GO: Gene Ontology
GPCR: G-Protein Coupled Receptor
AST: aspartate aminotransferase
ALT: alanine aminotransferase
SDF-1: stromal cell derived factor 1
